# Antibacterial activity of Thymoquinone, an active principle of *Nigella sativa *and its potency to prevent bacterial biofilm formation

**DOI:** 10.1186/1472-6882-11-29

**Published:** 2011-04-13

**Authors:** Kamel Chaieb, Bochra Kouidhi, Hanene Jrah, Kacem Mahdouani, Amina Bakhrouf

**Affiliations:** 1 Laboratoire d'Analyses, Traitement et Valorisation des Polluants de l'Environnement et des Produits, Faculté de Pharmacie, rue Avicenne 5000, Université Monastir, Monastir, Tunisia

## Abstract

**Background:**

Thymoquinone is an active principle of *Nigella sativa *seed known as "Habbah Al-Sauda" in Arabic countries and "Sinouj" in Tunisia. Bacterial biofilms tend to exhibit significant tolerance to antimicrobials drugs during infections.

**Methods:**

The antibacterial activity of Thymoquinone (TQ) and its biofilm inhibition potencies were investigated on 11 human pathogenic bacteria. The growth and development of the biofilm were assessed using the crystal violet (CV) and the 2, 3-bis [2-methyloxy-4-nitro-5-sulfophenyl]-2H-tetrazolium-5-carboxanilide (XTT) reduction assay.

**Results:**

TQ exhibited a significant bactericidal activity against the majority of the tested bacteria (MICs values ranged from 8 to 32 μg/ml) especially Gram positive cocci (*Staphylococcus aureus *ATCC 25923 and *Staphylococcus epidermidis *CIP 106510). Crystal violet assay demonstrated that the minimum biofilm inhibition concentration (BIC50) was reached with 22 and 60 μg/ml for *Staphylococcus aureus *ATCC 25923 and *Staphylococcus epidermidis *CIP 106510 respectively. In addition our data revealed that cells oxidative activity was influenced by TQ supplementation. In the same way, TQ prevented cell adhesion to glass slides surface.

**Conclusion:**

The ability of TQ to prevent biofilm formation warrants further investigation to explore its use as bioactive substances with antibiofilm potential.

## Background

A biofilm is a community of cells attached to biotic or abiotic surface [1,2]. It allows micro-organisms to survive in hostile environmental conditions [2]. Pathogenic bacteria released from the biofilm lead to food hygiene problems [3]. Conventional methods for biofilm removal are generally inadequate. Biofilm formation required the polysaccharide intercellular adhesion which contributed to cells protection against host immune system [4,5].

Prevention of biofilm formation effect of plants has been largely reported against *Listeria monocytogenes *[6], *Pseudomonas aeruginosa *[7], *Streptococcus mutans *[8-10], *Staphylococcus aureus *[11,12], *Candida albicans *[13] and oral pathogens [14]. The presence of rich biological active compounds in *Nigella sativa *volatile oil has highlighted its traditional medicinal use [15]. Black seed of *Nigella sativa *L. have been used in Middle Eastern folk medicine as a natural remedy for various diseases for over 2000 years [16]. Many active principles have been isolated from *Nigella sativa *seed [17] including thymoquinone (TQ). TQ (2-isopropyl-5-methyl-1,4-benzoquinone) was the bioactive constituent of this oil [18] showing antibacterial [19,20] and antifungal activity [21]. In addition a great antibacterial action of TQ against *Paenibacillus larvae *was observed (MIC values ranging from 8 to 16 mg/ml) [22]. Alkharfy et al., [23] reported that TH treatment reduced mortality in mice following Lipopolysaccharid and live *Esherichia coli *challenge by 80-90%. More recently, TQ inhibits the proliferation of MCF-7/DOX cells [24].

This study was undertaken to investigate the *in vitro *antibacterial activity of TQ and its potency to prevent biofilm formation against human pathogenic bacteria.

## Methods

### Organisms and chemicals

In this study, the antibacterial activity of TQ was tested on 11 Human pathogenic strains including Gram negative bacilli: *Escherichi coli *ATCC 35218, *Salmonella enterica *serovar Typhimurium ATCC 14028, *Pseudomonas aeruginosa *ATCC 27853, *Vibrio alginolyticus *ATCC 33787, *Vibrio paraheamolyticus *ATCC 17802; Gram positive bacilli: *Bacillus cereus *ATCC 14579, *Listeria monocytogene *ATCC 19115 and Gram positive cocci: *Enterococcus faecalis *ATCC 29212, *Micrococcus luteus *NCIMB 8166, *Staphylococcus aureus *ATCC 25923, *Staphylococcus epidermidis *CIP 106510 (Table 1).

**Table 1:** Antibacterial activity of thymoquinone against Human pathogenics strains

Strains	Antimicrobial susceptibility					
	
	Gentamycin (μg/ml)		Erythromycin (μg/ml)		Thymoquinone (μg/ml)	
	
	^**a**^**MIC**	^**b**^**MBC**	MIC	MBC	MIC	MBC
Gram negative bacilli						
*Escherichi coli *ATCC 35218	8	16	32	64	>512	>512
*Pseudomonas aeruginosa *ATCC 27853	2	4	256	>256	>512	>512
*Salmonella enterica *serovar Typhimurium ATCC 14028	2	8	>256	>256	>512	>512
*Vibrio alginolyticus *ATCC 33787	32	64	>256	>256	256	>512
*Vibrio paraheamolyticus *ATCC 17802	8	16	128	256	32	64
Gram positive bacilli						
*Bacillus cereus *ATCC 14579	4	8	8	16	8	8
*Listeria monocytogene *ATCC 19115	2	4	1	4	16	32
Gram positive cocci						
*Enterococcus faecalis *ATCC 29212	32	64	256	>256	32	64
*Micrococcus luteus *NCIMB 8166	2	8	4	16	8	64
*Staphylococcus aureus *ATCC 25923	16	32	16	32	8	16
*Staphylococcus epidermidis *CIP 106510	4	8	16	32	8	8

a, Minimum inhibitory concentration.

b, Minimum bactericidal concentration.

TQ, gentamycin and erythromycin was purchased from Sigma (Sigma-Aldrich, Switzerland).

### Minimum inhibitory concentration determination

The broth microdilution method was used to determine the minimum inhibitory concentration (MIC) and minimum bactericidal concentration (MBC) of TQ (0 to 512 μg/ml), gentamycin (0 to 256 μg/ml) and erythromycin (0 to 256 μg/ml) as recommended by the National Committee for Clinical Laboratory Standards Institute [25].

An overnight culture (37°C) of the tested strains were diluted 10-fold in fresh tryptic soy broth (TSB) and incubated (37°C) until they reached exponential growth phase. Serial two-fold dilutions of TQ in Mueller Hinton (MH) Broth (Biorad, France) were prepared in a 96-wells plate (190 μL per well).

The inocula (10 μL) containing 5. 10^6 ^cfu/ml of each reference strain were added to each well and the tested compound. A number of wells were reserved in each plate to test the sterility control of the medium (no inoculum added) and inoculum viability (no compound added).

After incubation for 24 h at 37°C, bacterial growth was evaluated by the presence of turbidity and a pellet on the well bottom. The MIC was defined as the concentration that completely inhibited visible cell growth during a 24-h incubation period at 37°C

### Minimum bactericidal concentration determination

To determine the minimum bactericidal concentration (MBC) values, 10 μL of each well medium with no visible growth was removed and inoculated in MH plates. After 24 h of incubation at 37°C, the number of surviving organisms was determined. MBC was defined as the lowest concentration at which 99% of the bacteria were killed. Each experiment was repeated at least twice [26].

### Effect of Thymoquinone on biofilm formation

#### Crystal Violet assay

TQ was tested for its potential to prevent biofilm formation of four reference strains (Table 2). The TQ was added to the growth medium at the time of inoculation and the cells were allowed to form biofilms [6]. Prevention of biofilm formation by TQ was examined by microdilution, similar to the MIC assay for planktonic cells. A two-fold serial dilution was prepared in 96-well polystyrene tissue culture plates containing TSB broth with 2% glucose (w/v), with final concentrations of TQ ranging from 0 to 512 μg/ml.

**Table 2:** Antibiofilm effect of thymoquinone against four positive biofilm strains

Strains	Inhibition of biofilm development (%)			
	
	Crystal Violet assay		XTT assay	
	
	^**a**^**BIC50 (μg/ml)**	^**b**^**BIC90 (μg/ml)**	BIC50 (μg/ml)	BIC90 (μg/ml)
*Enterococcus faecalis *ATCC 29212	85	349	44	145
*Staphylococcus aureus *ATCC 25923	22	75	20.5	51
*Staphylococcus epidermidis *CIP 106510	60	109	40	90
*Pseudomonas aeruginosa *ATCC 27853	>512	>512	>512	>512

a, minimum biofilm inhibition concentration of TQ that showed 50% inhibition on the biofilm formation.

b, minimum biofilm inhibition concentration of TQ that showed 90% inhibition on the biofilm formation.

The medium without TQ was used as the non-treated well and the medium with TQ as the blank control. Aliquots of bacterial suspension (10 μl) were inoculated in tissue culture plate wells (5.10^4 ^cfu/ml, final concentration). Following incubation at 37°C for 24h, culture supernatants from each well were decanted and planktonic cells were removed by washing three times with phosphate-buffered saline (7 mM Na_2_HPO_4_, 3 mM NaH_2_PO_4 _and 130 mM NaCl at pH 7.4). Cells in biofilm were fixed with methanol during 15 min, air dried and stained with 1% crystal violet [27]. Biofilm formation was quantified by measuring the absorbance at 595 nm using a microplate reader (GIO. DE VITA E C, Italy).

In order to asses the ability of TQ to prevent biofilm formation, the percentage of biofilm inhibition was calculated using the equation [(OD growth control ^_ ^OD sample)/OD growth control] × 100 [6]. Each assay was repeated three times.

The minimum biofilm inhibition concentration (MBIC50) was defined as the lowest concentration of TQ that showed 50% inhibition on the biofilm formation.

### Assessment of biofilm metabolic activity using XTT reduction assay

The metabolic activity of cells in biofilm was assessed using the XTT [2, 3-bis (2-methyloxy-4-nitro-5-sulfophenyl)-2H-tetrazolium-5-carboxanilide] reduction assay according to methods described previously [6,28] which measures the reduction of a tetrazolium salt by metabolically active cells to a coloured water soluble formazan derivative that can be easily quantified colorimetrically.

A two-fold serial dilution of TQ (final concentrations from 0 to 512 μg/ml) was prepared in 96-well polystyrene tissue culture plates containing TSB broth with 2% glucose (w/v). Than the plates were inoculated in the same way as described for crystal violet assay.

XTT (Sigma-Aldrich, Switzerland) solution (1 mg/ml) was prepared in PBS, filter sterilized and stored at -80°C. Menadione (Sigma-Aldrich, Switzerland) solution (1 mM) was prepared in acetone and sterilized immediately before each assay.

Following incubation, the biofilms were first washed five times with PBS, and then 100 μl PBS and 12 μl XTT-menadione solution (12.5:1 v/v) were added to each of the prewashed wells and the control wells. The plate was then incubated for 3 h in the dark at 37°C. Following incubation, 100 μl of the solution was transferred to fresh wells, and the colour change in the solution was measured with a multiskan reader at 492 nm. The absorbance values for the controls were then subtracted from the values of the tested wells to eliminate spurious results due to background interference.

The percentage of biofilm inhibition was calculated using the equation [(OD growth control ^_ ^OD sample)/OD growth control] × 100. Each assay was repeated three times.

### Microscopic techniques

Prevention of biofilm formation by TQ was confirmed by microscopic technique. Briefly, strains were allowed to grow on round covers glass slides (diameter 1 cm) placed in 24-well polystyrene plates (Greiner Bio-One, France) supplemented with TQ (0, MIC, 2 × MIC), incubated for 24 h at 37°C and stained with 1/20 Giemsa (Sigma, Switzerland) solution (v/v) for 20 min at room temperature. Stained glass pieces were placed on slides with the biofilm pointing up and were inspected by light microscopy at magnifications X100.

### Statistical analysis

Statistical analysis was performed on SPSS v.17.0 statistics software. Statistical differences and significance were assessed by one-way ANOVA test and Wilcoxon signed ranks test, as appropriate, to evaluate the biofilm inhibition according the type of strains and the TQ supplementation. A *P *value < 0.05 was considered significant.

## Results

### Effect of thymoquinone on viability of planktonic cells

TQ demonstrated selective antimicrobial properties. As presented in table 1, it exhibited bactericidal activity on 7 out of 11 tested strains with MIC and MBC values ranging from 8 to 32 μg/ml and 8 to 64 μg/ml, respectively. This activity is nearly similar to the tested antibiotics (gentamycin and erythromycin). However, Gram negative bacilli (*Escherichia coli *ATCC 35218, *Salmonella enterica *serovar Typhimurium ATCC 14028, *Pseudomonas aeruginosa *ATCC 27853), seem to be resistant to TQ action (MIC and MBC > 512 μg/ml). We noted also that the MBC values of TQ were 2-4 times higher than the MICs values.

### Inhibition of biofilm formation

#### Crystal violet assay

Prevention of biofilm formation by TQ was tested on four positive strains (Table 2). Results were expressed as inhibition percentages of biofilm development. TQ showed a significant inhibitory effect (*P *< 0.05) on biofilm formation of *Staphylococcus epidermidis *CIP 106510 and *Staphylococcus aureus *ATCC 25923 with a dose dependent manner.

As presented in table 2, the lower BIC50 of TQ was observed for *Staphylococcus aureus *ATCC 25923 (22 μg/ml), followed by *Staphylococcus epidermidis *CIP 106510 (60 μg/ml) and *Enterococcus feacalis *ATCC 29212 (85 μg/ml).

Our results demonstrated that TQ induced prevention of 90% of biofilm formation of *Staphylococcus aureus *ATCC 25923, *Staphylococcus epidermidis *CIP 106510 and *Enterococcus faecalis *ATCC 29212 when used at 75, 109 and 349 μg/ml respectively, suggesting that its strong biofilm inhibition potencies is not restricted to staphylococci. However, our data showed also that TQ do not prevent 50% of biofilm formation in the case of *Pseudomonas aeruginosa *ATCC 27853.

### Effect of thymoquinone on biofilm oxidative activity

In the presence of TQ, the metabolic oxidative activity of cells in biofilms was distinctly reduced after 24 h of incubation (Table 2). Our data also provides preliminary evidence that TQ affect the oxidative activity of all the tested strains compared to the non treated biofilm (Table 2).

The BIC50 was observed with TQ concentration about 20.5, 40 and 44 μg/ml for *Staphylococcus aureus *ATCC 25923; *Staphylococcus epidermidis *CIP 106510; and *Enterococcus faecalis *ATCC 29212 respectively (Table 2). Moreover, BIC90 was very low (51, 90 and 145 μg/ml) suggesting that TQ is efficient for prevention of biofilm formation. We noted also that *Pseudomonas aeruginosa *ATCC 27853 was less susceptible to TQ than the others strains. A statistical significant difference in prevention of biofilm formation between the treated strains with TQ (> 4 μg/ml) and control was found (*P *< 0.001). These results indicated that in addition to reducing the number of adherent bacteria assessed by crystal violet assay, TQ has an effect on the metabolic activity of cells embedded in biofilm.

### Prevention of biofilm formation on glass microscope slide covers

**Figure 1:**
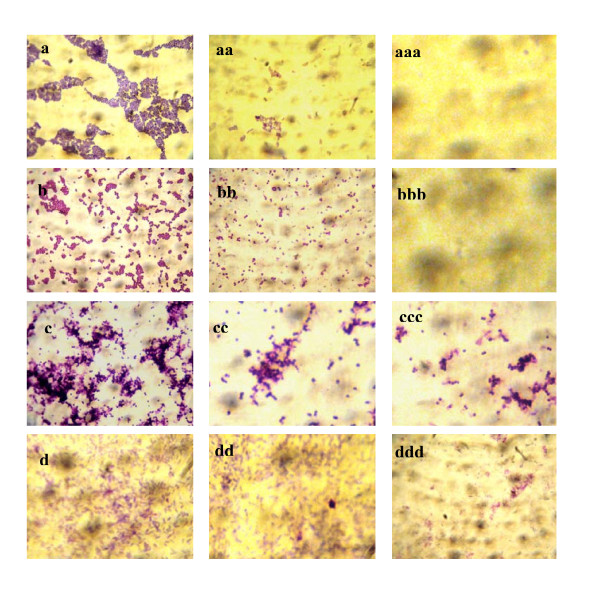
**Microscopic visualization of the effect of thymoquinone on four biofilm positives strains cultured on glass slides covers**. Prevention of biofilm formation effect of TQ was as followed: For *S. aureus *ATCC 25923 a, non treated slides; aa, cells supplemented with TQ MIC; aaa, cells supplemented with TQ 2 × MIC. For *S. epidermidis *CIP 106510, b, positive control (non treated slides); bb, cells supplemented with TQ MIC; bbb, cells supplemented with TQ 2 × MIC.For *E. faecalis *ATCC 29212, c, non treated slides; cc, cells supplemented with TQ MIC; ccc, cells supplemented with TQ 2 × MIC. For *P. aeruginosa *ATCC 27853, d, non treated slides; dd, cells supplemented with TQ MIC; ddd, cells supplemented with TQ 2 × MIC.

Prevention of biofilm formation by TQ was confirmed by microscopic visualization. As shown in figure 1, a moderate reduction of biofilm formation was observed with TQ supplementation (1 MIC) on the strong biofilm formers (*Staphylococcus aureus *ATCC 25923 and *Staphylococcus epidermidis *CIP 106510) whereas the biofilm former was significantly inhibited with 2 × MIC TQ supplementation. With this last concentration, the biofilm former of *Enterococcus faecalis *ATCC 29212 and *Pseudomonas aeruginosa *ATCC 27853 decreased but was not wholly suppressed.

## Discussion

Based on our present results, TQ exhibited a selective antibacterial effect against seven bacteria, particularly Gram positive strains with low MICs values (Table 1). This result correlate with Kokoska et al., [29] who reported that Thymoquinone exhibited potent growth-inhibitory effect against Gram-positive bacteria, with MICs ranging from 8 to 64 μg/ml.

Bacteria in biofilm have been shown to be much more resistant to antibiotics than their planktonic form [30]. The success of natural compounds in inhibiting cell attachment is a promising tool for reducing microbial colonization on various surfaces [31]. Application of anti-adhesion agents appears to be a very interesting approach in the prevention of microbial infection [32,33].

In order to find a natural compound able to inhibit and prevent microbial biofilm formation, we tested the effect of TQ on four biofilm positives strains. Crystal violet assay showed that TQ reduce the number of adherent bacteria and the BIC50 was reached with 22 and 60 μg/ml for *Staphylococcus aureus *ATCC 25923 and *Staphylococcus epidermidis *CIP 106510 respectively (Table 2).

We noted also that the medium supplemented with 75 μg/ml of TQ induce 90% biofilm inhibition in the case of *Staphylococcus aureus *ATCC 25923 (Table 2). For *Enterococcus feacalis *ATCC 29212 and *Pseudomonas aeruginosa *ATCC 27853, BICs90 was higher than 100 μg/ml.

A statistically significant inhibitory effect on biofilm formation by *Staphylococcus aureus *ATCC 25923 and *Staphylococcus epidermidis *CIP 106510 was noted after TQ supplementation (*P *< 0.001).

Prevention of biofilm formation by TQ was also confirmed using XTT assay. At 51 μg/ml TQ supplementation, it exhibited a significant biofilm inhibition percentage of *Staphylococcus aureus *ATCC 25923 that was more than 90% (Table 2). In addition BIC90 was reached with 90 and 145 μg/ml supplementation for *Staphylococcus epidermidis *CIP 106510 and *Enterococcus faecalis *ATCC 29212 respectively (Table 2). The Wilcoxon signed ranks test showed a statistical significant difference between the none treated and the treated cells with concentrations over 4 μg/ml (*P *< 0.001). Most antibiotics are up to 1000-times less efficient against bacteria in biofilm than in suspension [34], which makes TQ a very promising treatment alternative.

Inhibition of biofilm formation assessed by XTT do not correlate with crystal violet assay, similar result has been reported for plant extracts between the biomass and metabolic activity [6].

Our results revealed that TQ efficiently kills staphylococci in suspension and prevent biofilms formation. This effect on biofilm formation was confirmed by microscopic analysis of strains grown on the surface of glass slides covers. We observed a biofilm inhibition when we inoculated the strain with a concentration of TQ equal to MIC and 2 × MIC. Statistical analysis revealed a significant difference between the percentage of biofilm inhibition obtained after TQ supplementation (2 × MIC) between treated cells and non treated ones (*P *< 0.001).

## Conclusion

TQ significantly affects pathogenic bacteria at low concentrations. Its antimicrobial and biofilm inhibition potencies allows us to suggest its inclusion in the arsenal of bioactive substances and subjecting it to further research, such as *in vivo *compatibility tests in many biological models. However, further work needs to be done to determine the main mechanism by which TQ affect biofilm formation.

## Competing interests

The authors declare that they have no competing interests.

## Authors' contributions

KC was the primary author of the manuscript, assisted in antimicrobial assay, minimum inhibition concentration determination, antibioflms assay of Thymoquinone. BK was the person contributed in antibioflms assay and helped in the writing of the manuscript. HJ was the person participated in data acquisition and contributed in writing of the manuscript. KM designed and planned the study, and participated in the writing of the manuscript. AB provided funding, supervised the study, and helped to finalize the manuscript. All the authors read and approved the final version of the manuscript.

## Pre-publication history

The pre-publication history for this paper can be accessed here:

http://www.biomedcentral.com/1472-6882/11/29/prepub
